# Identification by Molecular Docking of Homoisoflavones from *Leopoldia comosa* as Ligands of Estrogen Receptors

**DOI:** 10.3390/molecules23040894

**Published:** 2018-04-12

**Authors:** Fedora Grande, Bruno Rizzuti, Maria A. Occhiuzzi, Giuseppina Ioele, Teresa Casacchia, Fabrizio Gelmini, Rita Guzzi, Antonio Garofalo, Giancarlo Statti

**Affiliations:** 1 Department of Pharmacy, Health and Nutritional Sciences, University of Calabria, Ampl. Polifunzionale, Via P. Bucci, 87036 Rende (CS), Italy; mariaantonietta.occhiuzzi@unical.it (M.A.O.); giuseppina.ioele@unical.it (G.I.); teresa.casacchia@unical.it (T.C.); antonio.garofalo@unical.it (A.G.); giancarlo.statti@unical.it (G.S.); 2 CNR-NANOTEC, Licryl-UOS Cosenza and CEMIF.Cal, Department of Physics, University of Calabria, Via P. Bucci, 87036 Rende (CS), Italy; rita.guzzi@fis.unical.it; 3 Department of Environmental Science and Policy–ESP, University of Milan, Via Celoria 2, 20133 Milan, Italy; fabrizio.gelmini@unimi.it; 4 Department of Physics, University of Calabria, Via P. Bucci, 87036 Rende (CS), Italy

**Keywords:** natural compounds, molecular docking, estrogen receptors, ligand interactions

## Abstract

The physiological responses to estrogen hormones are mediated within specific tissues by at least two distinct receptors, ERα and ERβ. Several natural and synthetic molecules show activity by interacting with these proteins. In particular, a number of vegetal compounds known as phytoestrogens shows estrogenic or anti-estrogenic activity. The majority of these compounds belongs to the isoflavones family and the most representative one, genistein, shows anti-proliferative effects on various hormone-sensitive cancer cells, including breast, ovarian and prostate cancer. In this work we describe the identification of structurally related homoisoflavones isolated from *Leopoldia comosa* (L.) Parl. (*L. comosa*), a perennial bulbous plant, potentially useful as hormonal substitutes or complements in cancer treatments. Two of these compounds have been selected as potential ligands of estrogen receptors (ERs) and the interaction with both isoforms of estrogen receptors have been investigated through molecular docking on their crystallographic structures. The results provide evidence of the binding of these compounds to the target receptors and their interactions with key residues of the active sites of the two proteins, and thus they could represent suitable leads for the development of novel tools for the dissection of ER signaling and the development of new pharmacological treatments in hormone-sensitive cancers.

## 1. Introduction

The estrogen receptors (ERs), members of the nuclear receptor family, are ligand-inducible intracellular transcription factors and are involved in the regulation of several physiological processes, including cell growth, survival and differentiation [1,2,3]. In mammals, cellular responses to estrogens are mainly mediated by two different estrogen receptor subtypes, ERα and ERβ, which exhibit variable expression and distribution levels, as well as distinct signaling responses. ERα is predominantly expressed in female reproductive organs, breast, kidney, bone, white adipose tissue and liver, whereas ERβ has been found in several tissues of both male and female bodies including the central nervous system, colon, lung, kidney, male reproductive organs, and cardiovascular and immune systems. As members of the nuclear receptor protein family, ERs are located in the nucleus, even if they are also present in the cytoplasm and mitochondria [4].

The activities of ERs are modulated by a variety of natural and synthetic ligands. The activated ligand-ER homodimer complex binds to specific DNA sequences EREs (estrogen-response elements) and regulates transcription activity throughout interactions with specific transcription modulators. In some cases, ERα and ERβ could form heterodimers, and experimental evidence suggests that in this form they can induce activation of target genes that are different from those induced by homodimers [5,6,7,8].

**Figure 1:**
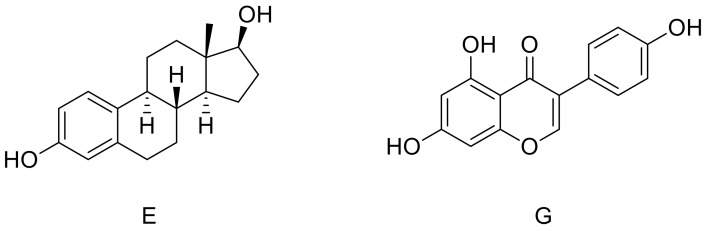
Structures of 17β-estradiol (**E**) and genistein **(G)**.

Extensive data [9,10,11,12,13] have been recently published on the ER structures, their intra- and inter-molecular interactions, and related post-translational modifications. These proteins are the natural target of estrogens, such as 17β-estradiol (**E**) (Figure 1), which are steroid hormones biosynthesized initially from cholesterol. ERα is expressed only in a low fraction of cells in healthy breast epithelium, whereas its expression significantly increases (up to 80% of cells) in breast cancer [14]. On the other hand, more than 80% of normal breast epithelial cells express ERβ, whereas its presence is reduced or completely suppressed during breast cancer emergence and progression [15]. Accordingly, anti-hormonal therapy is commonly adopted for the treatment of breast cancer in patients that show an over-expression of such a receptor.

A number of chemical compounds derived from plants, known as phytoestrogens, demonstrates the ability to bind to the estrogen receptors, producing estrogenic or anti-estrogenic activity. Their potential anti-proliferative effects could be useful for the formulation of nutraceuticals or pharmaceutics. Tamoxifen (**TX**), a selective estrogen-receptor modulator (SERM), is nowadays the most effective and widely used anti-estrogen drug used for this purpose. However, several patients treated with **TX** develop a rapid onset of resistance [4]. Thus, the identification of novel anti-estrogen compounds able to overcome resistance onset is still demanding.

Several natural compounds have shown interesting estrogenic activity, the majority of them belonging to the isoflavones family. The most representative one, genistein (**G**) (Figure 1), has shown anti-proliferative effects on various hormone-sensitive cancer cells including breast, ovarian and prostate cells [16,17,18]. The structure of **G** has many points of similarity to **E,** and this could explain its capability in binding the estrogen receptors within the same active site.

The increasing interest of the scientific community in the health benefits of the Mediterranean diet led us to investigate the estrogenic activity of homoisoflavones structurally related to **G** and isolated from *Leopoldia comosa* (L.) Parl., (ex Muscari), a perennial bulbous plant that is endemic in southern Italy. A number of ethnobotanical investigations demonstrated a wide use of *L. comosa* bulbs in traditional cooking recipes and in the formulation of remedies useful for the treatment of toothache and skin spots [19,20,21]. Earlier studies demonstrated the anti-oxidant, anti-inflammatory, diuretic, and anti-obesity properties of *L. comosa* extracts mainly due to the considerable presence of homoisoflavones [22,23,24,25]. These natural anti-oxidants were also found endowed with cytotoxic properties in a panel of in vitro human cancer models at low micromolar-range concentrations [22,26].

This study describes the identification of two homoisoflavones isolated from *L. comosa* as ER ligands, thus useful as hormonal substitutes or complements in breast cancer treatments. Molecular docking studies on the crystallographic structures of ERs have been carried out to investigate the binding mode of these homoisoflavones on both receptors. The results indicate that the two novel molecules can effectively bind ERs in the same binding site of both **E** and **G**. Furthermore, they show a greater conformational adaptability in their binding geometry compared to **G**, within both ERα and ERβ, thus proving that they could be of interest for the development of selective agents useful in the treatment of hormone-sensitive cancers.

## 2. Results and Discussion

### 2.1. Homoisoflavones from Leopoldia comosa

**Figure 2:**
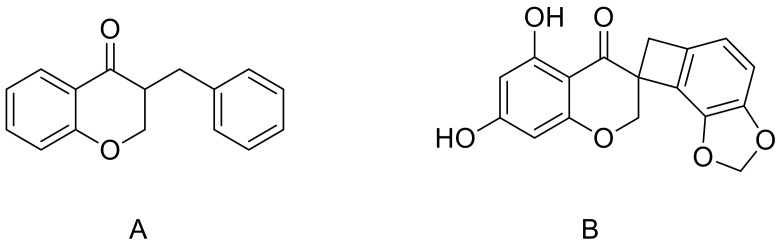
Structures of 3-benzylchroman-4-one (**A**) and scillascillin (**B**).

Several phenolic compounds based on a 3-benzylchroman-4-one skeleton and termed homoisoflavones have been isolated from various genres of the Hyacinthaceae plants family [27]. These compounds are generally classified in three small groups: 3-benzyl-4-cromanones, 3-benzylidene-4-cromanones and scillascylinoid homoisoflavones [28,29]. Although these compounds are considered as a sub-class of the extensively studied flavonoids, some steps in their biosynthetic pathways still need to be elucidated and, therefore, further studies are necessary to fully establish their chemical origin and biological properties [30]. Homoisoflavonoids are biosynthesized from cinnamic acid derivatives along with malonyl-CoA sub-units through the shikimato route by following the polyketide pathway. In a following step, phenylalanine is formed from prephenate and leads to a C-4, C-3, C-9 backbone, to which another ring is added from the acetate/mevalonate pathway [31]. Geopedological aspects and agronomic techniques have a significant influence on the polyphenol content and related biological activity of the plant extracts [32]. In the genus *L.*, two different classes of homoisoflavonoids have been identified: 3-benzylchroman-4-one containing compounds and scillascillin derivatives (Figure 2).

**Figure 3:**
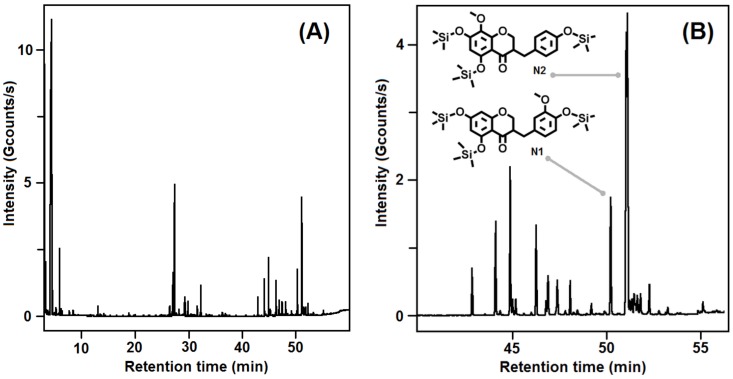
Gas chromatography–mass spectrometry (GC–MS) analysis of the hydroalcoholic extract of *L. comosa* bulbs: (**A**) chromatographic profile (total ion current, TIC); (**B**) detail of the chromatogram including indication of the peaks of the two silyl-functionalized homoisoflavones **N1** and **N2**.

**Figure 4:**
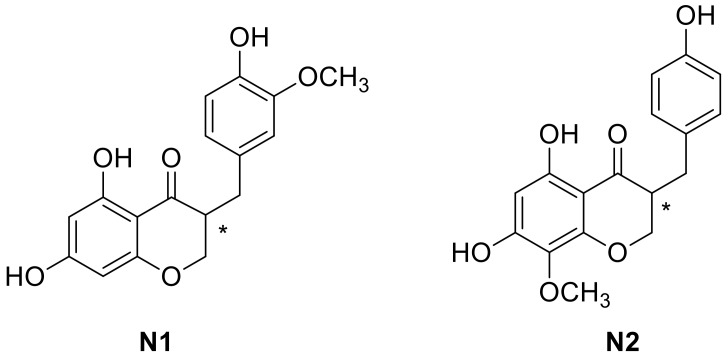
Structures of the identified homoisoflavones **N1** and **N2**.

In this study, bulbs of *L. comosa* were collected in the fields of the Sila Massif, Calabria, southern Italy. The bulbs were opportunely stored in a cool and dry environment and successively separated from roots and soil residues. The bulbs were then subjected to extraction with a water/ethanol mixture. The total phenol and the total flavonoid content of the whole extract was determined using the Folin–Ciocalteu reagent and chlorogenic acid as standard [33] and with the AlCl_3_ colorimetric method [34], respectively, following known procedures [23]. The phytochemical identification of homoisoflavones present in the total extract of the bulbs was conducted through semi-preparative high-performance liquid chromatography (HPLC) and gas chromatography–mass spectrometry (GC–MS) (Figure 3), after functionalization of the whole extract. Comparison of the results obtained with the two applied techniques allowed the recognition of two compounds, **N1** (5,7-dihydroxy-3-(4-hydroxy-3-methoxybenzyl)chroman-4-one) and **N2** (5,7-dihydroxy-3-(4-hydroxybenzyl)-8-methoxychroman-4-one), belonging to the 3-benzylchroman-4-one class (Figure 4).

### 2.2. Binding to Estrogen Receptor (ER) Proteins

Molecular docking is a useful technique to investigate the association of ligands to a target protein, and it has already been applied to elucidate details of the binding properties of ERs [35,36,37,38]. Accordingly, after identification of the two naturally occurring homoisoflavones **N1** and **N2**, molecular docking studies were performed to investigate their binding to the active site of both ERs. To this aim, the binding modes and affinities of these two compounds were compared to those obtained for known ligands of ERs, **E** and **G.** In particular, we investigated whether the replacement of the appended substituted phenyl ring of **G** with a substituted benzyl of **N1** and **N2** was consistent with a retained affinity within the active site of the receptors. Such an alteration confers a higher degree of flexibility to the whole molecular asset and, on the other hand, keeps aromatic moieties slightly farther from each other with respect to their position in **G**. As a result, the calculated distance between 3- and 17-OH of **E** and the corresponding groups in **G** (7- and phenolic-OH) remains almost unchanged between 7-OH and phenolic-OH in **N1** and **N2**. Furthermore, the presence of a stereocenter in both compounds (Figure 4) led to enantiomers, which could differently accommodate into the receptor’s active site. In order to evaluate the influence of chirality to the interaction with the key residues of the active site of ERs, molecular docking studies were performed on both enantiomers of each compound.

The availability of several ER models allowed us to pursue a structure-based approach to characterize the interaction of isolated homoisoflavones within the ligand-binding pocket of the receptors. The ligands were docked into the crystallographic structures of both ERα and ERβ, obtained from the Protein Data Bank (PDB) [39]. The crystallographic structures selected for this study include three ERα-**E** and two ERα-**G** complexes, as well as one ERβ-**E** and two ERβ-**G** complexes. In some of these complexes the receptor is represented as a monomer (1X7R [40] and 2OCF [41] for ERα, and 1QKM [42] for ERβ) and in other as homodimer (2QA8 [43], 1A52 [44] and 1GWR [45] for ERα, and 1X7J [40] and 5TOA [46] for ERβ).

In a first step, the crystallographic structures of both ERs in complex with **E** and **G** were considered, and their shape complementarity, hydrogen-bonding network, and van der Waals clashes with the ligands were thoroughly analyzed. The crystal structures of the receptors showed the presence of a compact ellipsoid cavity, where ligands are surrounded by a hydrophobic environment. Within this pocket, OH groups of **E** at positions 3 and 17, as well as the hydroxyl groups of **G** at position 7 and phenolic-OH and a further OH group at position 5, play an important role in orienting the ligand within the active site.

For evaluating the binding affinity of the ligands in their anchoring location, the scoring function of AutoDock Vina [47] was used with a re-docking of the crystallographic binding poses in their exact crystallographic position, without performing any search within the protein volume. The resulting binding energies, reported in Table 1, ranged from −9.4 to −10.0 kcal/mol for **E** (−9.7 kcal/mol on average), and from −7.8 to −9.6 kcal/mol for **G** (−8.9 kcal/mol on average). These findings point to a preference in the binding of **E** compared to **G**, although the variability obtained in the binding values of the latter is higher (standard deviations were 0.2 and 0.7 kcal/mol for **E** and **G**, respectively). In contrast, no significant differences were found between ERα and ERβ in the binding values for these two ligands.

**Figure 5:**
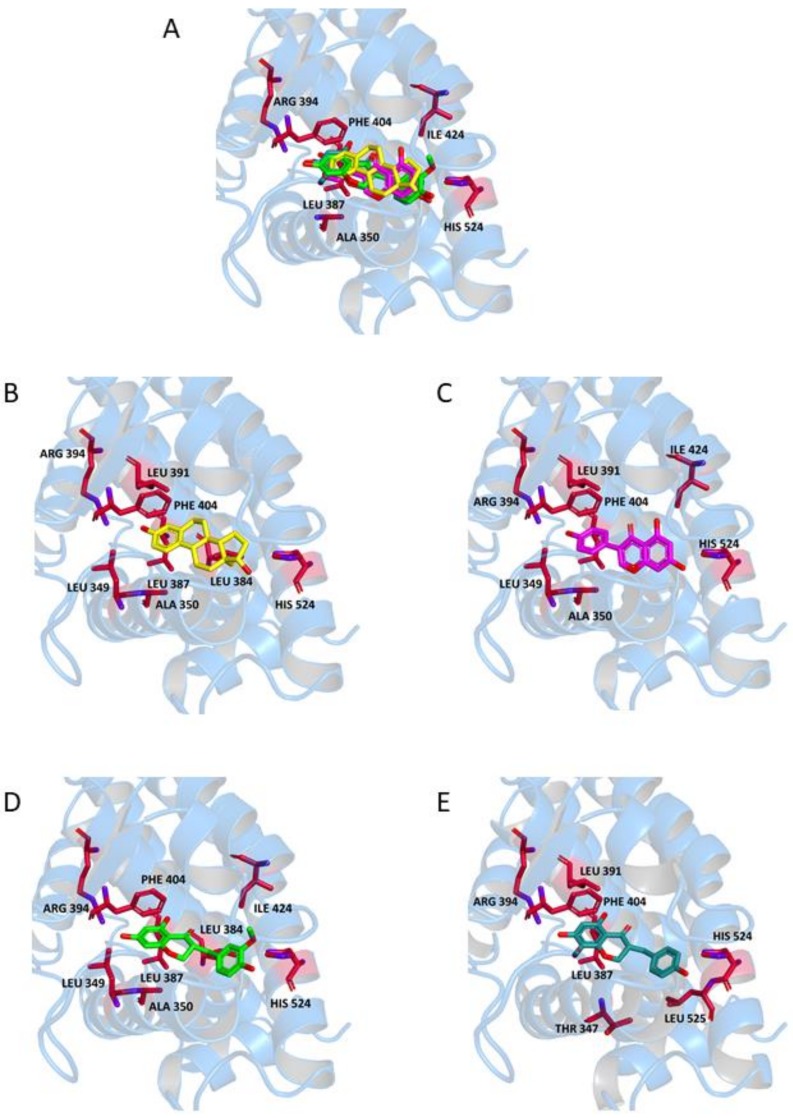
Ligand-binding pocket of the active site of ERα; ribbons representing protein structural elements are also shown. (**A**) Superimposed binding modes of all the four ligands: **E** (yellow), **G** (magenta), **(R)-N1** (green), and **(R)-N2** (cyan); the key residues are also indicated in the specific binding mode of (**B**) **E**; (**C**) **G**; (**D**) **(R)-N1**; and (**E**) **(R)-N2**.

**Figure 6:**
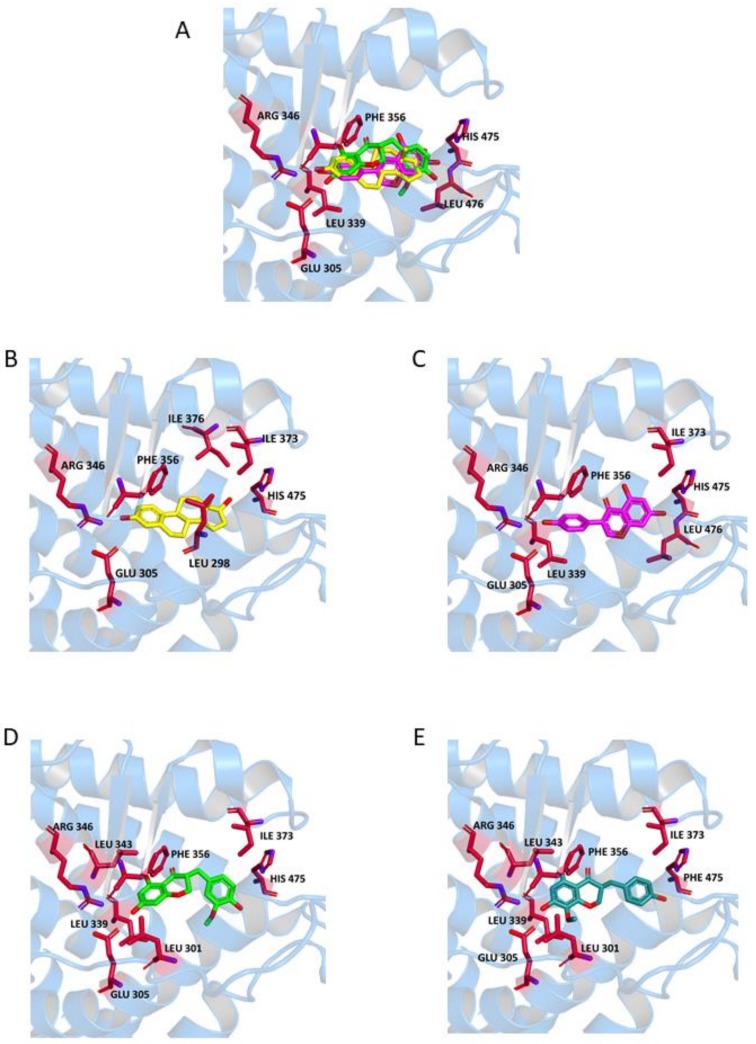
Ligand-binding pocket of the active site of ERβ; ribbons representing protein structural elements are also shown. (**A**) Superimposed binding modes of all the four ligands: **E** (yellow), **G** (magenta), **(R)-N1** (green), and **(R)-N2** (cyan); the key residues are also indicated in the specific binding mode of (**B**) **E**; (**C**) **G**; (**D**) **(R)-N1**; and (**E**) **(R)-N2**.

We also tested the ability of the docking engine to identify the ligand-binding site in a blind search performed on the protein volume. To this end, docking simulations were carried out with the unliganded receptors (i.e., the protein structures with the ligand taken away), considering again the binding of the two crystallographic ligands. As shown in Figure 5 and Figure 6, both **E** and **G** were found to bind within the ER site in the same position determined in crystallography, with a root mean square displacement ≤1 Å. The complexes obtained were further refined in an energy minimization procedure by using a molecular mechanics protocol [48] to mimic the refinement process commonly adopted in crystallography [49,50] and for a more direct comparison with the protocol that we later used for testing the binding of our compounds (see below).

The affinities obtained for **E** and **G** (see Table 1) were on average −9.8 and −8.8 kcal/mol, respectively, confirming a preference in the binding of the former ligand by 1 kcal/mol. The deviation in the variability of the binding energies were 0.3 kcal/mol for both ligands, proving that the energetic preference in the binding of **E** is significant. As in the case of the re-docking of the crystallographic ligands without performing any search, differences between ERα and ERβ in the binding of these ligands were found to be negligible. We also noted that, although the binding energies found with the two procedures (i.e., either re-docking the binding modes in their exact crystallographic position or performing a blind search followed by an energy minimization) were not identical in general, their average value and dispersion were the same. Thus, the values obtained for **E** and **G**, reported above, can be considered as references to compare the binding affinity of other compounds to the same receptors.

After assessing the binding of known ligands to the receptors, we proceeded in the estimate of the binding properties of our new compounds. The results of molecular docking performed using the crystallographic structures of the ERα and ERβ showed in all cases association of the compounds within the same binding sites as **E** and **G**. These binding modes were obtained for the ligand docked to rigid receptors, and did not include any re-accommodation of the protein structure. Thus, the complexes obtained were further refined with the same energy-minimization process previously used in the case of the blind docking of the crystallographic ligands. The binding scores found at the end of the refinement procedure are reported in Table 2. The binding energy values were −8.8 ± 0.3, −8.6 ± 0.4, −8.6 ± 0.4 and −8.7 ± 0.6 kcal/mol for **(R)-N1**, **(S)-N1**, **(R)-N2** and **(S)-N2**, respectively. These values are similar to those obtained in the binding of the crystallographic ligand **G**, i.e., −8.9 ± 0.7 and −8.8 ± 0.3 kcal/mol in both cases previously discussed (i.e., without or with blind search, respectively). These results strongly suggest that our compounds are able to accommodate in both enantiomeric forms into the active site of ERs with the same affinity of **G**, and with only a slightly lower affinity compared to **E**.

We completed our analysis by investigating the protein residues involved in the ligand-receptor binding within the active site of the two types of receptors in the case of homoisoflavones **N1** and **N2**. Resulting interactions are reported (Table 3) for both the (R) and (S) enantiomers, and are compared with those established by ligands **E** and **G** in the binding sites of the crystallographic structures. It can be clearly seen that not only the binding energies are similar for the two enantiomers of both **N1** and **N2** (see Table 2), but also the interacting protein residues are the same with only a few exceptions (indicated with the labels ′(R)′ and ′(S)′ in Table 3). This observation confirms that our ligands are able to bind both ERs with a larger variety of conformational possibilities compared to **E** and **G**.

Visual inspection provided further details on the binding modes of **N1** and **N2** within the ERs; due to the similarities of the binding energies and anchoring residues discussed above for their two enantiomers, in the following we will show models representing only the (R) form, although the discussion will regard both forms. In the binding pocket of ERα (Figure 5, Panels A–E), both 17-OH of **E** and 7-OH of **G** interact with His524 through formation of a hydrogen bond. An equivalent interaction is formed by the phenolic-OH group of **N1** and **N2**. Moreover, 7-OH of **N1** and **N2** is involved in further hydrogen bonds with Arg394 and Leu387, similarly to the 3-OH of **E** and the phenolic-OH of **G**. Additional stabilization of the complex results from a π-stacking interaction between Phe404 and the aromatic ring of **E** or the appended phenyl of **G**. The same interaction could be established between the aromatic amino acid and the benzo-fused ring of **N1** and **N2**. The ligand-receptor complex is further stabilized for **N1** and **N2** by hydrophobic interactions with Ala350, Ile424, and a number of Leu residues.

In ERβ (Figure 6), all ligands are involved in a hydrogen bond with His475 and a π-stacking interaction with Phe356. In particular, an interaction with Hys475 is established by 17-OH of **E**, 7-OH of **G** and the phenolic-OH of **N1** and **N2**. A π-stacking interaction with Phe356 involves the aromatic ring of **E** or the appended phenyl of **G**. Similarly to ERα, this interaction is established by the benzo-fused moiety of **N1** and **N2**. For this receptor, a hydrogen bond formation was observed between Arg346 and the 3-OH of **E**, the phenolic-OH of **G**, and the 7-OH of **N1** and **N2**. The ligand-receptor complex is further stabilized in the presence of **N1** and **N2** by hydrophobic interactions with Leu301 and Leu339, along with some other interactions specific for each ligand. The presence of the extra carbon atom between the two cyclic portions of the molecule in **N1** and **N2** is compatible with their accommodation into the active site of both receptors, although the two fused ring systems of **N1** and **N2** are in a reversed orientation with respect to **G**.

In summary, our data suggest that the described compounds are sterically compatible and form binding interactions with the key residues of the active site of both ERα and ERβ. It is interesting to note that, besides the conformations of **N1** and **N2** described above, which closely resemble those of the crystallographic ligand **E** and **G**, we also observed some alternative orientations of our ligands with slightly lower or even comparable energies. This observation is perhaps not surprising, since **N1** and **N2** possess a larger conformational freedom compared to the structure of both **E** and **G**. Although this possibility does not affect our docking results in terms of binding energy, it could be exploited in the use of these homoisoflavones as leading compounds for further optimization, with the aim of designing ligands with enhanced affinity toward ERs.

## 3. Materials and Methods 

### 3.1. Plant Material and Phytochemical Profile

Bulbs of *L. comosa* were collected in the fields of the Sila Massif (39°40′27.34′′ N, 16°46′79.24′′ E–39°40′9.20′′ N, 16°46′84.91′′ E), Calabria, southern Italy. The bulbs were stored in a cool and dry environment and subsequently separated from roots and cleaned of soil residues. The bulbs (580 g) were used for an extraction with a water/ethanol mixture (1:1 *v*/*v*) using a Naviglio ® extractor (Atlas Filtri S.r.L., Limena, PD, Italy) according to previously reported procedures [23]. The total phenol content of the whole extracts was determined using Folin–Ciocalteu reagent and chlorogenic acid as a standard [33]. The total flavonoid content of the crude extract was determined by the AlCl_3_ colorimetric method on the same extracts used for total phenol determination [34]. The phytochemical composition of the hydroalcoholic extract was investigated by high-performance liquid chromatography coupled to ultraviolet diode array detection (HPLC–UVDAD) and by gas chromatography coupled with mass spectrometric detection (GC–MS) after derivatization (silanization) of the sample as previously described [23].

### 3.2. Molecular Docking 

Eight crystallographic structures of ERs [40,41,42,43,44,45,46] including five in dimeric form, were obtained from the PDB, and consisted of the protein in complex with either **E** or **G**. The structures of both **E** and **G** were extracted from the crystallographic complexes, while the structures of **N1** and **N2**, considered in either (R) or (S) structural conformations, were built by using the modeling software Avogadro [52]. Molecular docking was performed by using AutoDock Vina 1.1.2 (the Scripps Research Institute, La Jolla, CA, USA) [47]. A preliminary conversion of the structures from the PDB format was performed by using the graphical interface AutoDock Tools 1.5.6 (the Scripps Research Institute, La Jolla, CA, USA) [53]. During the conversion, polar hydrogens were added for the crystallographic ligands, and apolar hydrogens of **N1** and **N2** were merged to the carbon atom they are attached to. To account for the binding in any possible internal pocket of ERs, a search volume including the whole protein was considered, with a grid space of 1 Å.

Full flexibility was guaranteed to all the ligands, resulting in two active torsions for **E** (the two hydroxyls) and four for **G** (three hydroxyls, plus the bond connecting the phenol group to the rest of the molecule). Six rotations around dihedral angles were allowed for both **N1** and **N2** molecules. A single run was carried out at very high exhaustiveness (16 times larger than the default value) in each case. Re-docking experiments were also performed as score-only assessment without any search. Refinement of the complex structures was performed by energy minimization using the web server AMMOS2 [48], which employs the universal force field (UFF) potential set [54] and AMBER partial charges [55] with a conjugate gradient optimization. Intermolecular interactions were evaluated by using the automated protein–ligand interaction profiler (PLIP) [51].

## 4. Conclusions

In this work we have investigated the capability of newly identified homoisoflavones to form complexes with ER proteins. Our results confirm that these compounds are sterically compatible to accommodation within the active site occupied by previously known ligands, **E** and **G**, and interact with the same key protein residues of both the isoforms of ERs. Because of the known anti-tumor effect of the parent compound, i.e. the isoflavone **G**, and due to the possibility of using other natural homoisoflavones or employing these compounds as lead drugs for optimization through a rational design, this study could be considered the starting point for the identification of novel tools in the treatment of estrogen-sensitive breast-cancer. Further studies will be performed successively in order to assess the agonistic or antagonistic activity for these homoisoflavone compounds.

## Figures and Tables

**Table 1:** **Binding energies for E and G complexed with ERα** **and ERβ.** Energetic evaluation is performed by using the scoring function of AutoDock Vina [47], either without any search (score-only assessment for the crystallographic poses of the ligands) or exploring the whole receptor (volume-search on the protein structure) followed by a minimization refinement for the complex obtained. Results for receptors in dimeric form are reported with two values corresponding to chain A and B, respectively.

Protein Data Bank (PDB) Entry	Receptor	Ligand	Binding Energy (kcal/mol)	
			Score-Only	Volume-Search
1A52	ERα	**E**	−9.7/−9.8	−9.5/−9.8
1GWR	ERα	**E**	−9.7/−10.0	−10.3/−10.1
2OCF	ERα	**E**	−9.4	−9.7
2QA8	ERα	**G**	−8.6/−9.6	−8.7/−8.7
1X7R	ERα	**G**	−7.8	−8.5
5TOA	ERβ	**E**	−9.7/−9.9	−9.5/−9.5
1X7J	ERβ	**G**	−9.2/−8.6	−8.6/−8.8
1QKM	ERβ	**G**	−9.5	−9.4

**Table 2:** **Binding energies for N1 and N2 in both enantiomeric forms complexed with ERα** **and ERβ.** Docking performed by using AutoDock Vina [31], followed by a minimization refinement for the complex obtained. Results for receptors in dimeric form are reported with two values corresponding to chain A and B, respectively.

PDB Entry	Receptor	Binding Energy (kcal/mol)			
		(R)-N1	(S)-N1	(R)-N2	(S)-N2
1A52	ERα	−8.4/−8.5	−8.2/−8.2	−8.2/−8.9	−9.2/−9.3
1GWR	ERα	−8.4/−8.6	−8.3/−8.5	−8.6/−8.6	−8.9/−9.2
2OCF	ERα	−8.6	−8.9	−9.4	−8.6
2QA8	ERα	−8.8/−9.1	−8.9/−9.5	−8.8/−9.0	−8.0/−8.3
1X7R	ERα	−9.1	−8.4	−8.1	−8.5
5TOA	ERβ	−8.4/−8.6	−8.2/−8.4	−8.2/−8.7	−7.2/−8.7
1X7J	ERβ	−9.2/−9.2	−8.4/−8.9	−8.5/−8.7	−8.8/−9.0
1QKM	ERβ	−9.2	−9.0	−8.8	−8.8

**Table 3:** **Key protein residues of estrogen receptors (ERs) interacting with the ligands.** Interactions identified with protein–ligand interaction profiler (PLIP) [51]: HI = hydrophobic interaction; HB = hydrogen bond; π-st. = π-stacking. Interactions with residues in Italics are unique to only one out of the four ligands. (R) or (S) indicate that the interaction in solely formed by either of the two enantiomers of **N1**/**N2**.

	Interacting Residues					
Ligand	ERα			ERβ		
	HI	HB	π-st.	HI	HB	π-st.
**E**	Leu349 Ala350 Leu384 Leu387 Leu391	Leu387 Arg394 His524	Phe404	*Leu298* Ile373 Ile376	Glu305 Arg346 His475	Phe356
**G**	Leu349 Leu387 Leu391 Ile424	*Ala350* Leu387 Arg394 His524	Phe404	Leu339 Ile373 Leu476	Glu305 Arg346 His475	Phe356
**N1**	Leu349 (S) Ala350 (S) Leu384 Ile 424 Leu525 ^(*1)^ (R)	Leu387 Arg394 His524	Phe404	Leu301 ^(*2)^ (R) Leu339 *Leu343* ^(*3)^ Ile373 *His475* ^(*4)^ (S)	*Leu298* *Leu339* ^(*5)^ *Leu343* ^(*3)^ Arg346 His475	Phe356
**N2**	Leu349 (S) Leu387 Leu391 Leu525 ^(*1)^ (R)	*Thr347* Leu387 Arg394 His524	Phe404	Leu301 ^(*2)^ Leu339	Arg346 *Phe356* ^(*6)^ His475	Phe356

Notes: ^(*1)^ corresponds to Leu476 in ERβ that forms HI with **G**; ^(*2)^ corresponds to Leu349 in ERα that forms HI with all the ligands; ^(*3)^ corresponds to Leu391 in ERα that forms HI with **E**, **G** and **N2**; ^(*4)^ forms HI solely with **N1**, but forms HB with all the ligands; ^(*5)^ corresponds to Leu387 in ERα that forms HI with **E** and **G**; ^(*6)^ forms HI solely with **N2**, but forms HB with all the ligands.
